# Personalized Environment and Genes Study (PEGS) Dataset-a resource for genomic, exposomic, and geospatial data

**DOI:** 10.1038/s41597-026-07011-x

**Published:** 2026-04-02

**Authors:** Farida S. Akhtari, Jennifer Madenspacher, Diane D’Agostin, Samantha Shuptrine, Nathaniel MacNell, Rebecca Ritter, Adam Burkholder, Zongli Xu, Jasmine A. Mack, John S. House, Daniel J. Schaid, Shannon K. McDonnell, Shepherd H. Schurman, David C. Fargo, Charles P. Schmitt, Janet E. Hall, Alison A. Motsinger-Reif

**Affiliations:** 1 Biostatistics and Computational Biology Branch, National Institute of Environmental Health Sciences, Durham, NC USA; 2 Clinical Research Branch, National Institute of Environmental Health Sciences, Durham, NC USA; 3 DLH Corporation, Bethesda, MD USA; 4 Office of the Director, National Institute of Environmental Health Sciences, Durham, NC USA; 5 Department of Obstetrics and Gynaecology, University of Cambridge, Cambridge, UK; 6 Department of Quantitative Health Sciences, Mayo Clinic, Rochester, MN USA; 7 Clinical Research Core, National Institute on Aging, Bethesda, MD USA; 8 Office of Data Science, National Institute of Environmental Health Science, Durham, NC USA

**Keywords:** Data acquisition, Data integration, Genetic interaction

## Abstract

The Personalized Environment and Genes Study (PEGS) is a unique resource comprising genetic and environmental exposure data linked to geospatial data. The PEGS cohort contains 19,445 demographically diverse participants who provided phenotype and exposure data by completing three surveys. Whole-genome sequencing was performed for a subset of 4,737 participants to interrogate common and rare variants and structural variations, including high-resolution human leukocyte antigen (HLA) variants. Geographic coordinates were assigned to participant addresses, enabling the use of distance to contaminant sources and area-level air-pollutant concentrations as surrogates for exposure. Several available tools are available to explore these data and results of exposome-wide association studies (ExWAS) conducted in the data. The i2b2 Query and Analysis Tool enables approved users to build customizable queries for exploring basic statistics from de-identified and aggregated PEGS data. PEGS Explorer allows users to explore published ExWAS results and rigorously calculated exposure correlations. Globe visualizations in this tool reflect the complex mixtures involved in the exposome and allow users to visualize correlations between exposures and common, complex diseases.

## Background & Summary

Genetic and environmental factors interact in complex ways to influence individual-level health. Due to this intricate interplay, collecting and analyzing data on genetics and the environment are crucial for a comprehensive understanding of disease etiology and prevention tactics. Genetic data enables the identification of variations in an individual’s DNA that can affect disease susceptibility, providing insights into inherent risk factors, enabling personalized risk assessments, and informing early interventions. Combined with genetic data, environmental data is used to identify how genetic variation interacts with exposures, engendering a deeper understanding of how risk factors vary based on individual exposures.

Globally, an estimated 25% to 90% of overall disease etiology can be attributed to environmental factors^1–5^. Moreover, many diseases result from interactions between an individual’s genetic predisposition and their particular environmental exposures such as smoking or air pollution, highlighting the importance of considering both genetic and environmental factors. Integrating genetic and environmental data enables the development of personalized treatment plans and allows medications to be tailored to individual genetic profiles, leading to safer, more effective treatments with reduced adverse effects. Additionally, by understanding individual genetic and environmental risk factors, healthcare providers can offer personalized lifestyle change recommendations and suggest interventions aimed to prevent or mitigate disease risk.

While there is growing appreciation of the importance of including both genetic and environmental data in studies, the dearth of relevant datasets limits research opportunities. While whole-genome sequencing (WGS) data are available, collecting analogous exposome data is challenging. While exposome data are essential for elucidating the etiology of common, complex diseases, they are difficult to measure, collect, and compare. In this study, we present the Personalized Environment and Genes Study (PEGS), a unique data resource comprising genetic and extensive environmental exposure data available to the research community.

## Personalized Environment and Genes Study (PEGS)

PEGS collects survey-based exposomic, genomic, and geographic information system (GIS) data from a racially and ethnically diverse North Carolina-based cohort of nearly 20,000 individuals. The PEGS website summarizes the study and describes the available data (https://www.niehs.nih.gov/research/atniehs/labs/crb/studies/pegs/index.cfm). While most studies focus on a single disease or environmental exposure, PEGS collects data on multiple diseases and environmental exposures, including diet and lifestyle factors, in concert with genetic data. The goal of PEGS is to integrate these large-scale, multi-dimensional data to enable researchers to dissect the etiology of disease and identify the collective effects of environment, diet, lifestyle, and genetic factors on human health.

**Fig. 1:**
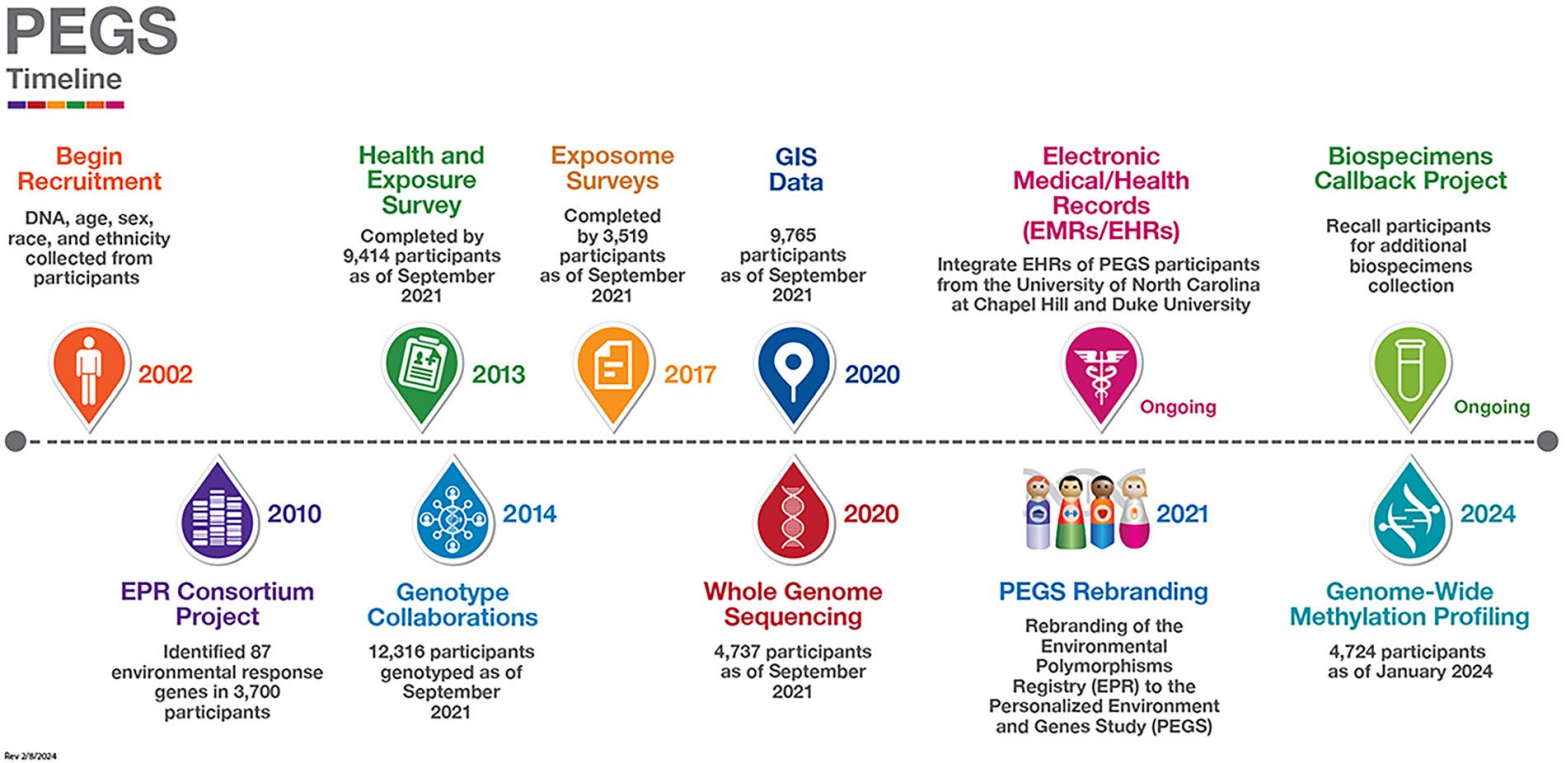
Timeline of the progression and expanded scope of the Personalized Environment and Genes Study (PEGS). Data reported from PEGS Data Freeze 3.1 created on 6/27/2023.

**Fig. 2:**
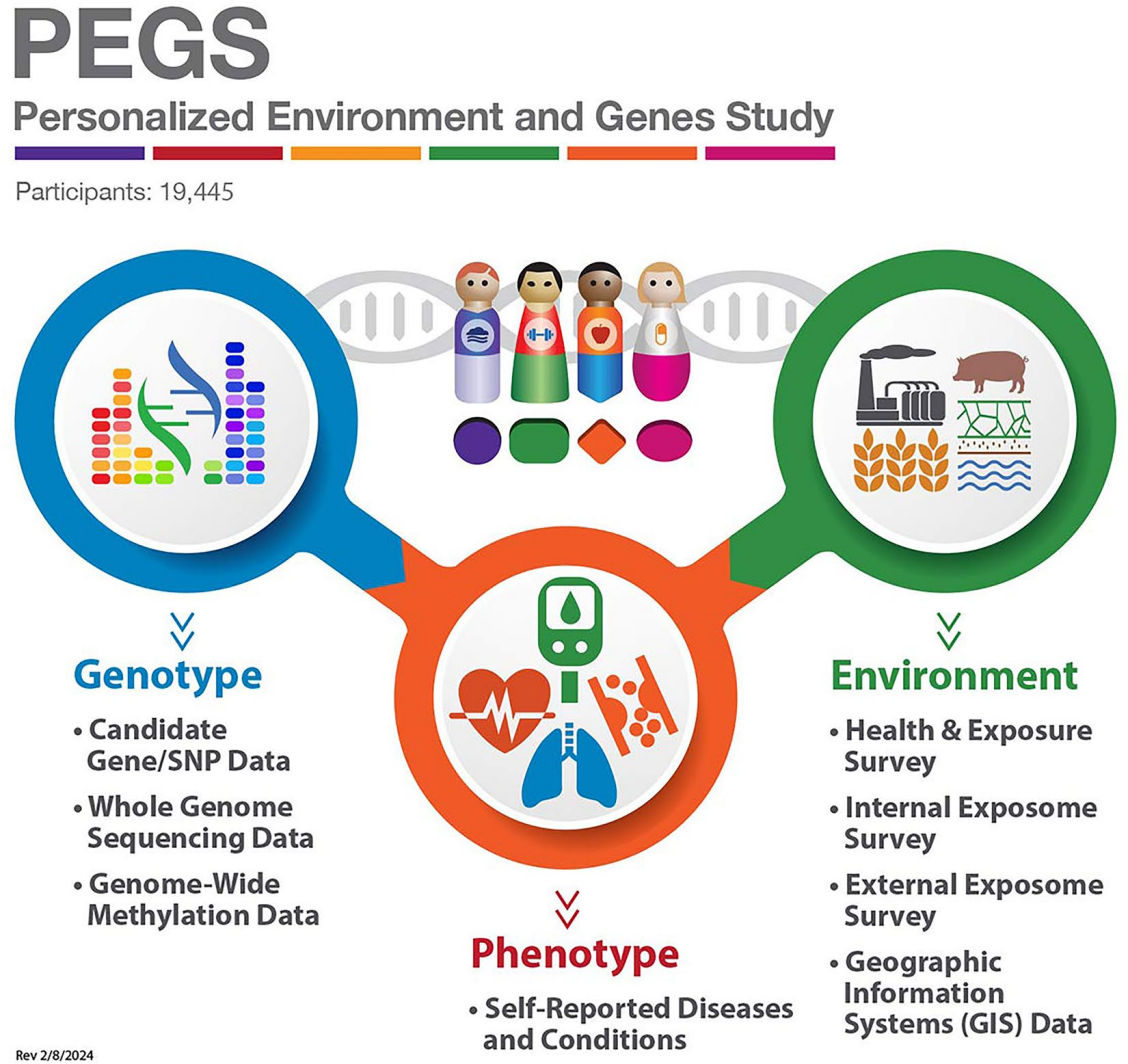
Overview of available PEGS data. Data reported from PEGS Data Freeze 3.1 created on 6/27/2023.

Originally established in 2002 for ongoing research at the National Institute of Environmental Health Sciences (NIEHS), the initial cross-sectional cohort of the Environmental Polymorphisms Registry (EPR) recruited participants by convenience sampling at community events. The EPR was renamed the Personalized Environment and Genes Study in 2022. Figure 1 is a timeline of the evolution of PEGS. PEGS represents a long-term investment by NIEHS in collecting and analyzing exposome data. The cohort has been expanded to function as an extensive repository of data on medication, lifestyle, and environmental exposures, diseases, and genetics, as shown in Fig. 2. Participants complete three surveys, the Health and Exposure Survey and the Internal and External Exposome Surveys, that request information on exposures at home and work and lifestyle factors such as sleep and diet. PEGS participants provide broad consent to share their data with researchers and data repositories for all relevant research. The National Institutes of Health Institutional Review Board (NIH IRB #: 04E0053) has approved the study protocol, informed consent forms, recruitment materials, all participant materials, and human data sharing, including genomic data sharing and publications. PEGS participants also consent to provide biological samples taken from multiple sites, including blood, skin, cheek, and nasal cells, exhaled breath, hair, nail clippings, saliva, sperm, sputum, stool, baby teeth, urine, and household dust. There is an option for participant call-back to collect additional tissue samples for specific studies with participant consent, including those collected during medical treatment or saved after biopsy or surgery.

### Study participants

**Fig. 3:**
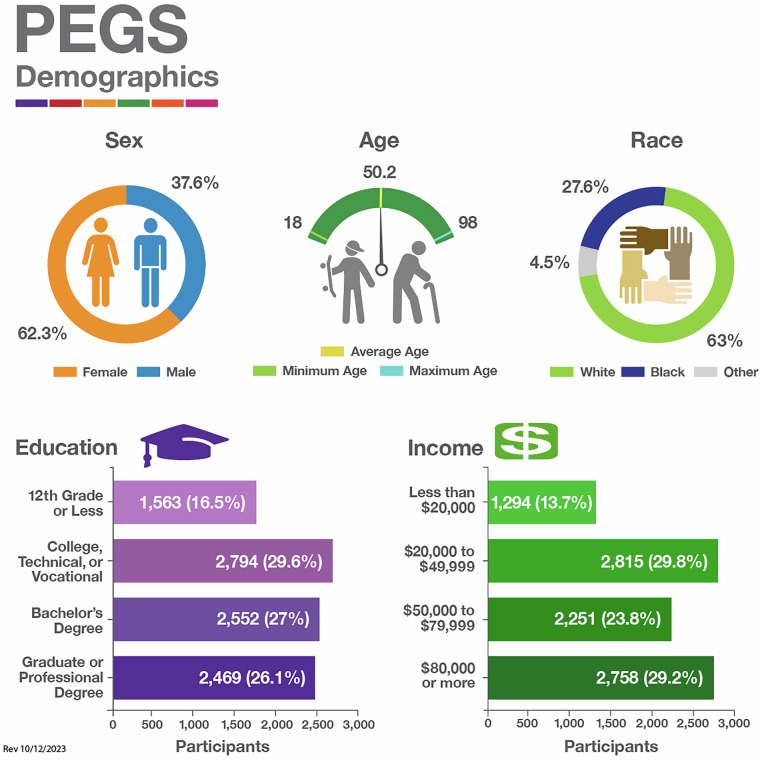
Demographics for PEGS participants, showing age at completion of the Health and Exposure Survey and self-reported sex, race, education level, and income level. Data reported from PEGS Data Freeze 3.1 created on 6/27/2023.

The 19,445 PEGS participants are demographically representative of the population of North Carolina. Table 1 provides demographic details, and Fig. 3 is a visual overview. Roughly two-thirds of participants are female (62.3%; 12,120), and one-third are male (37.6%; 7,298). Approximately two-thirds of participants self-identify as White (63%; 12,279), slightly over one-quarter self-identify as Black (27.6%; 5,361), and 4.5% self-identify as another race (868). Approximately 5% of participants self-identify as Hispanic (5.1%; 979). The cohort includes participants of varying education level, socioeconomic status, and age (range: 18.4–98.3 years), with a mean age of 50.2 years (at completion of the Health and Exposure Survey). Due to the diversity of the cohort, researchers can investigate disease risk in multiple populations and uncover health disparities across groups resulting from disproportionate environmental exposures. As a result, the findings from research using PEGS data are broadly applicable.

**Table 1:** PEGS participant demographics.

**Sex (N, %)**	
Female	12120 (62.3)
Male	7298 (37.5)
**Race (N, %)**	
White	12279 (63.1)
Black or African American	5361 (27.6)
Other	868 (4.5)
**Ethnicity (N, %)**	
Non-Hispanic/Non-Latino	17750 (91.3)
Hispanic/Latino	979 (5.0)
**Education (N, %)**	
Grade 12 or less	1563 (16.5)
College, technical, or vocational	2794 (29.6)
Bachelor’s degree	2552 (27.0)
Graduate or professional degree	2469 (26.1)
**Income (N, %)**	
Less than $20,000	1294 (13.7)
$20,000 to $49,999	2815 (29.8)
$50,000 to $79,999	2251 (23.8)
$80,000 or more	2758 (29.2)
**Age (mean, SD)**	50.2 (16.0)

Demographics of participants in the PEGS cohort. Age is at completion of the Health and Exposure Survey. Data reported from PEGS Data Freeze 3.1 created on 6/27/2023.

PEGS participants are recruited in outpatient clinics and hospitals and at over 200 unique sites in North Carolina that include businesses, career fairs, universities, community events such as charity walks, health fairs and festivals, senior centers, sporting events, social clubs, religious institutions, and military bases. Enrollment for the PEGS cohort is ongoing at the NIEHS Clinical Research Unit (https://www.niehs.nih.gov/research/atniehs/labs/crb/durham/index.cfm) in North Carolina, the PEGS enrollment website (https://joinastudy.niehs.nih.gov/studies/pegs), and Clinicaltrials.gov (ClinicalTrials.gov ID: NCT00341237).

Contact is maintained with over 6,000 PEGS participants. An important aspect of the cohort is the availability to recall participants to support additional data collection for follow-up studies to enable validation efforts, multi-omics data collection, and add-on studies. Through a collaboration with the National Human Genome Institute (NHGRI) Reverse Phenotyping Core, which has collected de-identified exome data from multiple investigators in the NIH intramural research program, researchers can contact participants for studies to determine the influence of genotypes of interest on individual phenotypes.

To protect the privacy, safety, and confidentiality of PEGS participants, PEGS data have not been deposited in a publicly available repository. The data are securely stored in a centralized, shared repository at NIEHS. To ensure the data are safely and securely available for research collaborations, de-identified versions of PEGS Data Freezes without personally identifiable information (PII) have been created for use when sharing PII or protected health information (PHI) is inappropriate and/or not required. PII or PHI was removed from all PEGS data components following the guidelines specified in the “NIST SP 800-122 Guide to protecting the confidentiality of personally identifiable information (PII)” by McCallister *et al*.^6^. Briefly, dates more specific than a year, participant address information more specific than state or territory, and participant identifiers that could be linked to health records or patient information were removed.

## Methods

### Questionnaire-based data

**Fig. 4:**
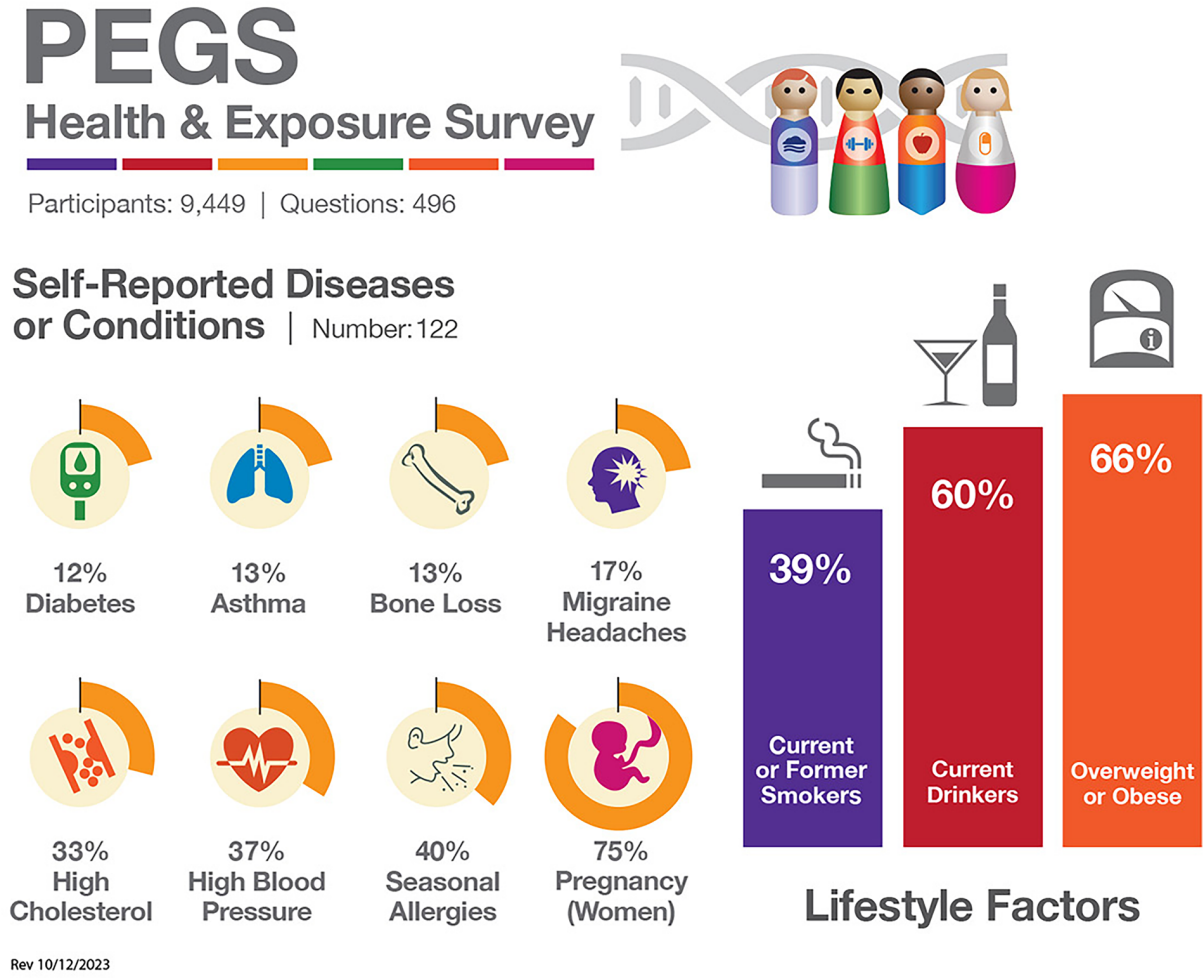
Percentages of participants with selected self-reported diseases or conditions and lifestyle factors from the PEGS Health and Exposure Survey. Data reported from PEGS Data Freeze 3.1 created on 6/27/2023.

**Fig. 5:**
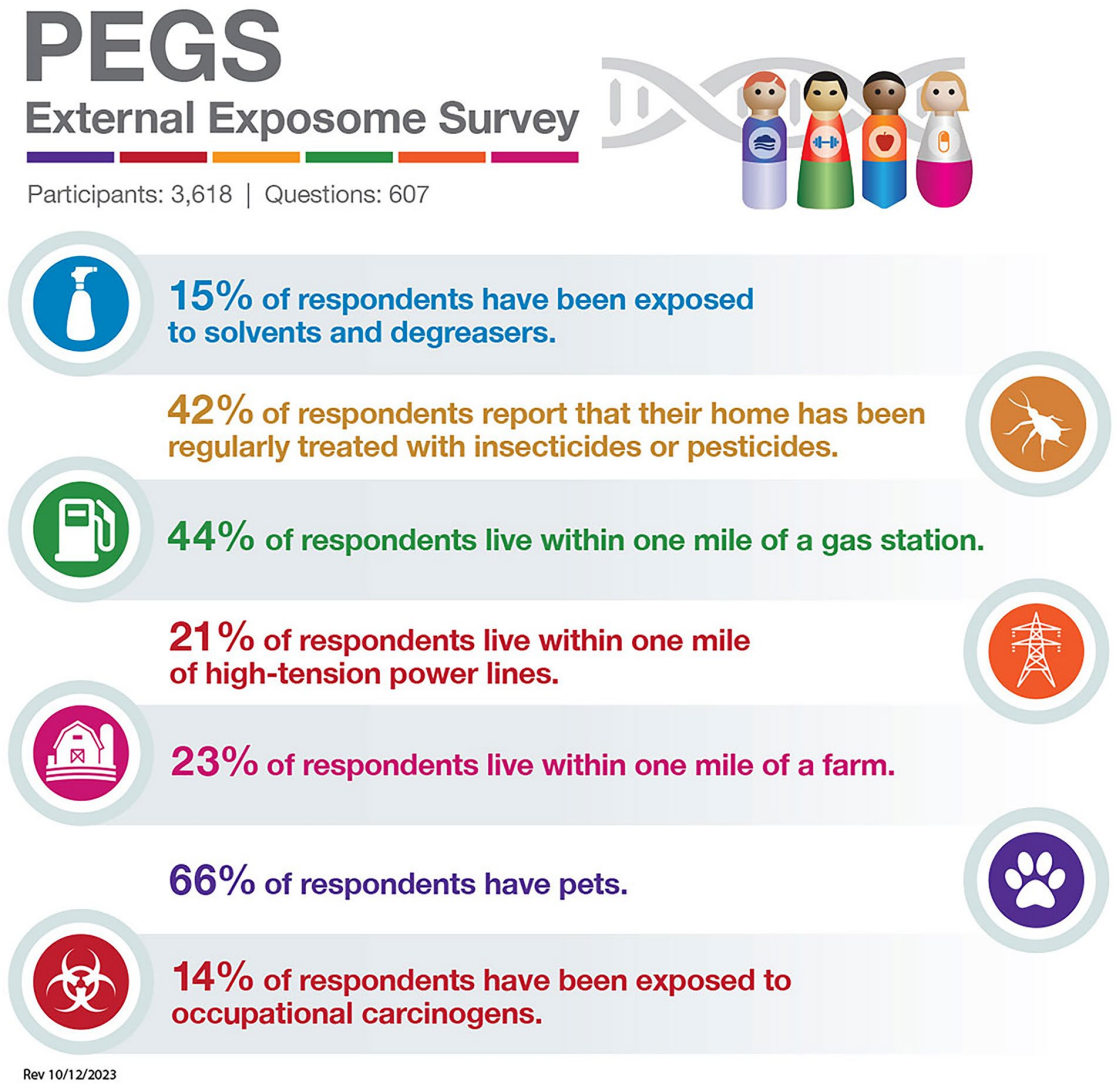
Percentages of participants exposed to selected external environmental exposures from the PEGS External Exposome Survey. Data reported from PEGS Data Freeze 3.1 created on 6/27/2023.

**Fig. 6:**
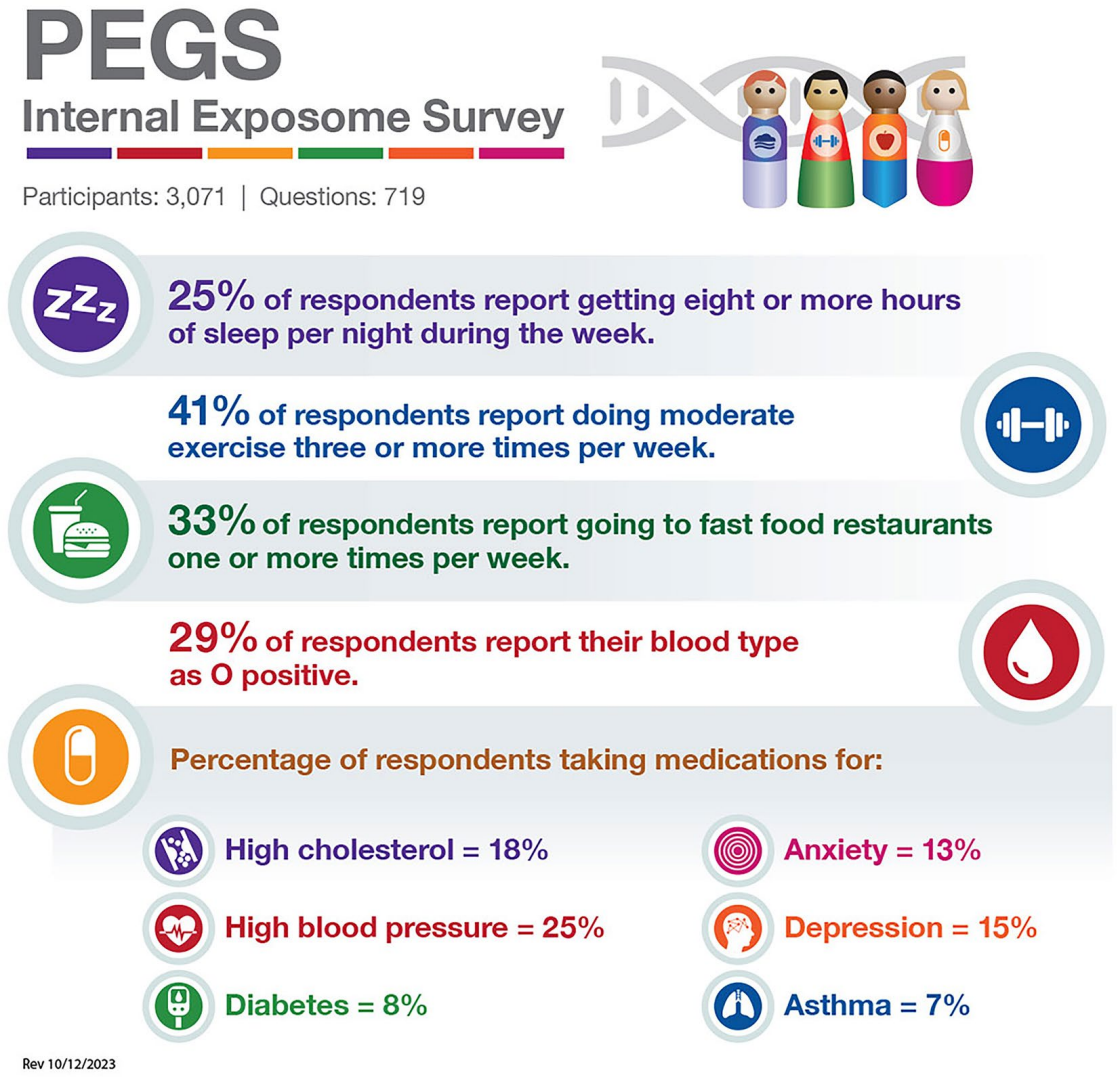
Percentages of PEGS participants exposed to selected internal environmental exposures from the PEGS Internal Exposome Survey. Data reported from PEGS Data Freeze 3.1 created on 6/27/2023.

PEGS participants complete three surveys to provide phenotype and exposure data. First administered in 2013, the PEGS Health and Exposure Survey (N = 9,449) collects data on general demographics, family medical history, lifestyle factors such as smoking and alcohol use, and occupational exposures. Beginning in 2017, the NIEHS PEGS Exposome Survey was also administered to collect comprehensive information about endogenous and exogenous exposures throughout life. The External Exposome Survey (Exposome Part A) (N = 3,618) is focused on external exposures, including chemical and environmental exposures at work and home from childhood to the present. The Internal Exposome Survey (Exposome Part B) (N = 3,071) is focused on internal exposures, including medications and lifestyle factors such as physical activity, stress, sleep, and diet. Table 2 outlines the question categories in the Health and Exposure Survey and the Internal and External Exposome Surveys, and Figs. 4–6 provide snapshots of the types of information requested by each survey. Additional screener surveys for diabetes and eczema were administered to 227 and 329 participants, respectively. The surveys are available for download on the PEGS website (https://www.niehs.nih.gov/research/atniehs/labs/crb/studies/pegs/about/data). Study protocol and other details can be found on Clinicaltrials.gov (ClinicalTrials.gov ID: NCT00341237).

**Table 2:** Categories of questions in the PEGS Health and Exposure Survey, External Exposome Survey, and Internal Exposome Survey.

**Health and Exposure Survey**				
About Your Family’s Health	Diabetes and Endocrine			Neurologic
About Your General Health	Digestive			Occupation
About Your Home Life	Exposures			Renal
About Your Mood	Fatigue			Reproductive (Females Only)
Bones, Joints, and Muscles	Hematological			Reproductive (Males Only)
Cancer	Immune			Respiratory
Cardiovascular	Lifestyle			Skin, Eyes, and Hair
**External Exposome (Exposome A)**				
Characteristics of Current and Past Residences: • Agricultural Property Use • Garage and Basement • Heating and Cooling • Pesticides and Insecticides • Pets • Surrounding Area • Walls and Flooring • Water and Dampness			Chemical and Metal Exposures at Work	
			Ultraviolet Light Exposure	
			Workplace Characteristics	
			Hobby Exposures	
**Internal Exposome (Exposome B)**				
Chemotherapy/Radiation Therapy		Physical Activity		
Dietary Behavior		Reproductive History (Females Only)		
Dietary Intake		Sleep		
Genetic History		Stress		
Infectious Disease		Vitamins, Minerals, and Other Supplement Use		
Medications		Twin/Triplet Siblings and Birth Order		
Other				

High-level survey question categories administered in the PEGS Health and Exposure Survey, External Exposome Survey, and Internal Exposome Survey. Data reported from PEGS Data Freeze 3.1 created on 6/27/2023.

Questionnaire data underwent structured quality control procedures, including range validation, logical consistency checks across related questions, verification of programmed skip patterns, and harmonization of categorical encodings across survey waves. Implausible responses were flagged based on predefined thresholds and reviewed. Free-text responses were standardized using rule-based parsing followed by manual review when necessary. Comprehensive data dictionaries and coding schemas accompany each PEGS Data Freeze to facilitate reproducible analysis. Self-reported data collected with the Health and Exposure Survey and the Exposome Surveys have been aggregated for case counts for numerous phenotypes. Tables 3 and 4 list self-reported phenotypes with a minimum of 100 cases among all participants and among participants for whom WGS data are available.

**Table 3:** Number of cases for self-reported diseases and conditions in PEGS participants.

Disease diagnosis	Cases-all participants	Disease diagnosis	Cases-all participants
Ever diagnosed with allergic rhinitis, hay fever, or seasonal allergies	3751	Cancer diagnosis - skin (non-melanoma)	508
Ever diagnosed with high blood pressure or hypertension	3460	Ever diagnosed with stomach or duodenal ulcer	502
Ever diagnosed with high cholesterol	3094	Diagnosed with manic-depressive illness or bipolar disorder	488
Ever diagnosed with allergies or allergic reactions (other than seasonal allergies)	2620	Ever diagnosed with rheumatoid arthritis (RA)	470
Ever diagnosed with chicken pox	2598	Ever diagnosed with chronic obstructive pulmonary disease (COPD) (e.g., chronic bronchitis, emphysema)	467
Ever diagnosed with flu	2062	Ever diagnosed with a sleep disorder	465
Ever diagnosed with migraine headaches (with or without aura)	1649	Males only - ever diagnosed with enlarged prostate or benign prostatic hyperplasia	460
Ever diagnosed with iron-deficiency anemia	1524	Ever diagnosed with viral food poisoning	458
Ever diagnosed with polyps in the colon or rectum	1469	Ever diagnosed with pyelonephritis, nephritis, or kidney infection	441
Females only - ever diagnosed with fibroids, fibroid tumors, uterine fibroids, or other benign uterine tumors	1457	Ever diagnosed with post-traumatic stress disorder (PTSD)	434
Any cancer diagnosis	1321	Ever diagnosed with a staph infection	432
Ever diagnosed with osteoarthritis	1247	Ever diagnosed with brittle bones or osteoporosis	426
Ever diagnosed with bone loss, thinning of the bones, osteopenia, or pre-osteoporosis	1240	Cancer diagnosis - had surgery	401
Ever diagnosed with asthma	1233	Ever diagnosed with psoriasis	399
Females only - ever diagnosed with ovarian cysts or benign ovarian growth or neoplasm	1224	Ever diagnosed with fibromyalgia	383
Ever diagnosed with diabetes or sugar diabetes	1142	Ever diagnosed with gout	364
Ever diagnosed with pre-diabetes, impaired fasting glucose, or impaired glucose tolerance	1126	Sleep disorder diagnosis - sleep apnea	362
Ever diagnosed with measles	1109	Females only - ever diagnosed with polyps in the endometrium or uterus	351
Ever diagnosed with thyroid disease (other than cancer)	1097	Ever diagnosed with shingles	350
Ever diagnosed with mumps	1000	Ever diagnosed with poor blood flow or blocked or narrowed arteries to the legs, claudication, or peripheral arterial disease	346
Ever diagnosed with cold sores	954	Ever diagnosed with diabetes - currently being treated with insulin	337
Ever diagnosed with another type of arthritis	912	Ever diagnosed with coronary artery disease	312
Ever diagnosed with eczema	877	Ever diagnosed with streptococcal invasive disease	297
Ever diagnosed with kidney stones	874	Ever diagnosed with heart attack or myocardial infarction (MI)	290
Ever diagnosed with shingles	834	Ever diagnosed with genital warts	275
Ever diagnosed with viral pneumonia	781	Cancer diagnosis - breast, including ductal carcinoma *in situ* (DCIS)	258
Ever diagnosed with German measles	773	Ever diagnosed with hepatitis	255
Ever diagnosed with gallbladder disease	752	Ever diagnosed with chlamydia	251
Ever diagnosed with urticaria or hives	734	Males only - ever diagnosed with inflammation of the prostate or prostatitis	242
Ever diagnosed with diabetes - currently using diabetic pills (oral medication) to lower blood sugar	729	Ever diagnosed with hyperthyroidism (e.g., Grave’s disease)	239
Ever diagnosed with lactose intolerance	712	Diagnosed with chronic fatigue syndrome by a doctor or health care professional	229
Ever diagnosed with hypothyroidism (e.g., Hashimoto’s thyroiditis)	703	Ever diagnosed with ulcers	220
Females only - ever diagnosed with endometriosis	661	Ever diagnosed with fatty liver disease or steatosis	214
Ever diagnosed with bacterial food poisoning	605	Ever diagnosed with Raynaud’s syndrome or disease	211
Ever diagnosed with cardiac arrhythmia	561	Ever diagnosed with enlarged thyroid or goiter	205
Ever diagnosed with bacterial pneumonia	534	Ever diagnosed with genital herpes	196
Ever diagnosed with mononucleosis (mono)	522	Ever diagnosed with whooping cough	196

Includes all diagnoses with at least 100 cases among all PEGS participants, arranged in decreasing order of number of cases among all participants. Data reported from PEGS Data Freeze 3.1 created on 6/27/2023.

**Table 4:** Number of cases for self-reported diseases and conditions in PEGS participants.

Disease diagnosis	Cases-participants with whole-genome sequencing	Disease diagnosis	Cases-participants with whole-genome sequencing
Ever diagnosed with allergic rhinitis, hay fever, or seasonal allergies	2050	Cancer diagnosis - skin (non-melanoma)	303
Ever diagnosed with high blood pressure or hypertension	1614	Ever diagnosed with stomach or duodenal ulcer	259
Ever diagnosed with high cholesterol	1617	Diagnosed with manic-depressive illness or bipolar disorder	188
Ever diagnosed with allergies or allergic reactions (other than seasonal allergies)	1427	Ever diagnosed with rheumatoid arthritis (RA)	193
Ever diagnosed with chicken pox	2070	Ever diagnosed with chronic obstructive pulmonary disease (COPD) (e.g., chronic bronchitis, emphysema)	189
Ever diagnosed with flu	1629	Ever diagnosed with a sleep disorder	374
Ever diagnosed with migraine headaches (with or without aura)	880	Males only - ever diagnosed with enlarged prostate or benign prostatic hyperplasia	243
Ever diagnosed with iron-deficiency anemia	827	Ever diagnosed with viral food poisoning	371
Ever diagnosed with polyps in the colon or rectum	816	Ever diagnosed with pyelonephritis, nephritis, or kidney infection	227
Females only - ever diagnosed with fibroids, fibroid tumors, uterine fibroids, or other benign uterine tumors	746	Ever diagnosed with post-traumatic stress disorder (PTSD)	208
Any cancer diagnosis	693	Ever diagnosed with a staph infection	341
Ever diagnosed with osteoarthritis	707	Ever diagnosed with brittle bones or osteoporosis	170
Ever diagnosed with bone loss, thinning of the bones, osteopenia, or pre-osteoporosis	666	Cancer diagnosis - had surgery	338
Ever diagnosed with asthma	607	Ever diagnosed with psoriasis	208
Females only - ever diagnosed with ovarian cysts or benign ovarian growth or neoplasm	660	Ever diagnosed with fibromyalgia	171
Ever diagnosed with diabetes or sugar diabetes	481	Ever diagnosed with gout	155
Ever diagnosed with pre-diabetes, impaired fasting glucose, or impaired glucose tolerance	567	Sleep disorder diagnosis - sleep apnea	291
Ever diagnosed with measles	920	Females only - ever diagnosed with polyps in the endometrium or uterus	192
Ever diagnosed with thyroid disease (other than cancer)	595	Ever diagnosed with shingles	282
Ever diagnosed with mumps	842	Ever diagnosed with poor blood flow or blocked or narrowed arteries to the legs, claudication, or peripheral arterial disease	136
Ever diagnosed with cold sores	771	Ever diagnosed with diabetes - currently being treated with insulin	118
Ever diagnosed with another type of arthritis	385	Ever diagnosed with coronary artery disease	141
Ever diagnosed with eczema	468	Ever diagnosed with streptococcal invasive disease	232
Ever diagnosed with kidney stones	429	Ever diagnosed with heart attack or myocardial infarction (MI)	112
Ever diagnosed with shingles	420	Ever diagnosed with genital warts	216
Ever diagnosed with viral pneumonia	606	Cancer diagnosis - breast, including ductal carcinoma *in situ* (DCIS)	139
Ever diagnosed with German measles	659	Ever diagnosed with hepatitis	129
Ever diagnosed with gallbladder disease	390	Ever diagnosed with chlamydia	184
Ever diagnosed with urticaria or hives	436	Males only - ever diagnosed with inflammation of the prostate or prostatitis	132
Ever diagnosed with diabetes - currently using diabetic pills (oral medication) to lower blood sugar	292	Ever diagnosed with hyperthyroidism (e.g., Grave’s disease)	119
Ever diagnosed with lactose intolerance	363	Diagnosed with chronic fatigue syndrome by a doctor or health care professional	100
Ever diagnosed with hypothyroidism (e.g., Hashimoto’s thyroiditis)	420	Ever diagnosed with ulcers	188
Females only - ever diagnosed with endometriosis	351	Ever diagnosed with fatty liver disease or steatosis	107
Ever diagnosed with bacterial food poisoning	485	Ever diagnosed with Raynaud’s syndrome or disease	134
Ever diagnosed with cardiac arrhythmia	308	Ever diagnosed with enlarged thyroid or goiter	100
Ever diagnosed with bacterial pneumonia	418	Ever diagnosed with genital herpes	146
Ever diagnosed with mononucleosis (mono)	427	Ever diagnosed with whooping cough	169

Includes all diagnoses with at least 100 cases among PEGS participants with available whole-genome sequencing data, arranged in decreasing order of number of cases among all participants. Data reported from PEGS Data Freeze 3.1 created on 6/27/2023.

Self-reported free-text data on medications collected with the Internal Exposome Survey have been translated into Anatomical Therapeutic Chemical (ATC) codes per the World Health Organization’s ATC classification system (https://www.who.int/tools/atc-ddd-toolkit/atc-classification). Briefly, the self-reported free-text medication data were cleaned and then matched to standardized medication names in the Food and Drug Administration’s National Drug Code Directory to find active ingredients for each listed medication name. These active ingredients were then matched to ATC codes from the 2021 ATC/DDD Index (https://www.whocc.no/atc_ddd_index/). Medications that did not match available standardized medication names were manually cleaned for spelling mistakes and regional differences in medication names or were encoded as supplements when appropriate. Drugs that did not match available ATC codes were included in a broader ATC code category when possible. The most complete level of ATC code and ATC name, if available, were mapped. A limitation of the questionnaire-based medication data is the unavailability of the route of administration and dosing. Table 5 outlines the data available for the PEGS cohort.

**Table 5:** PEGS data components.

Category and Components	Description	Participants
***Survey Data***		
**Demographic and administrative data**	Demographics, consent, address, and administrative data for all participants	19445
**Health and Exposure Survey**	Demographics, health, family history of disease, environmental exposures, socioeconomic status, and lifestyle	9449
**External Exposome Survey (Exposome A)**	Residential and occupational environmental exposures	3618
**Internal Exposome Survey (Exposome B)**	Medication use, physical activity, stress, sleep, diet, genetics, and reproductive history	3071
**Diabetes Screener Survey**	Diabetes screener administered to participants with self-reported diabetes	227
**Eczema Screener Survey**	Eczema screener administered to participants with self-reported eczema	329
**Right-not-to-know Main Survey**	Right-not-to-know survey administered for incidental findings and reports	231
**Right-not-to-know Cognitive Interview Survey**	Right-not-to-know cognitive interview administered to assess awareness of incidental findings and reports	12
***Medication Data***		
**Anatomical Therapeutic Chemical (ATC) codes**	ATC codes for self-reported free-text medication names from the Internal Exposome Survey (Exposome B) per the World Health Organization ATC classification system	2263
***Genomic Data***		
**Candidate gene/single-nucleotide polymorphism (SNP) data**	Candidate SNP data for a subset of participants for specific research goals	12316
**Single nucleotide variants (SNVs)**	SNV and small indel genotypes derived from the whole-genome sequencing (WGS) data in PLINK’s.bed/.bim/.fam format	4737
**Structural variants**	Structural variant calls generated from the whole-genome sequencing (WGS) data in.vcf format consisting of large deletions, duplications, and inversions	4737
**Human leukocyte antigen (HLA) genotypes**	HLA genotypes identified from the WGS data for 20 HLA genes with up to six digits of specificity	4737
**Telomeric content**	Aggregate telomeric content estimated from WGS reads reported as telomeric reads per GC content-matched million reads	4737
**Local and global ancestry estimations**	Inferred local ancestry per chromosome after haplotype phasing and global estimates of percent ancestry for each participant	4730
**Methylation data**	Genome-wide methylation profiling data using the Infinium MethylationEPIC v1.0 BeadChip Kit targeting 866,297 CpG sites	4724
***Geospatial Data***		
**Geocodes (GIS)**	Geocoded participant addresses from five study events with mapping coordinates	18462
**Hazards data**	Exposure estimates and proximity measures calculated using geospatial linkages from the following databases: Atmospheric Composition Analysis Group (ACAG), Toxics Release Inventory (TRI), Center for Air, Climate, and Energy Solutions (CACES), North Carolina Department of Environmental Quality (NCDEQ), Department of Transportation (DOT), Federal Aviation Administration (FAA), Federal Communications Commission (FCC), and the Nuclear Regulatory Commission (NRC)	18462
**MERRA-2 data (Earthdata)**	Geospatial data linkages from the Modern Era Retrospective Analysis for Research and Applications (MERRA-2) project containing consistent estimates of climate and environmental metrics from a range of satellite-based environmental observations	17273
**Social Vulnerability Index (SVI) data**	Geospatial data linkages for Centers for Disease Control/ Agency for Toxic Substances and Disease Registry (CDC/ATSDR) SVI containing summaries of social determinants of health at the census-tract level	17273
**Environmental Justice Index (EJI) data**	Geospatial data linkage for CDC/ATSDR EJI containing summaries of environmental, social, and health factors at the census-tract level	17273

Data components available for the PEGS cohort. Data reported from PEGS Data Freeze 3.1 created on 6/27/2023.

Self-reported income categories reflect nominal income at the time of survey completion and were not adjusted for inflation. Because specific survey dates were removed to protect participant privacy, users cannot retrospectively adjust income variables for inflation; analyses should interpret income categories as contemporaneous to survey completion.

### Whole-genome sequencing data

Initial genotyping of participant samples was completed as part of clinical call-back studies. In 2010, the pilot EPR Consortium Project genotyped 87 environmental response genes (656 single-nucleotide polymorphisms (SNPs) in approximately 3,700 randomly selected participants). Additionally, as part of genotype collaboration efforts initiated in 2014 to address specific research goals, genotyping was performed for 12,316 participants for targeted candidate genes and SNPs. In 2019, the Broad Institute performed WGS using blood samples from 4,737 PEGS participants with the most complete survey data to interrogate common and rare variants and structural variations, including high-resolution human leukocyte antigen (HLA) variants. Paired-end Illumina short-read sequencing was utilized for this purpose with a target depth of greater than 30x. The WGS data were aligned to the hg38 human reference assembly to obtain single-nucleotide variants and small insertions/deletions (indels). After quality control, approximately 43 million high-quality variants for 4,607 participants were annotated with the WGS Annotator. WGS also identified HLA genotypes with up to six digits of specificity for four- and six-digit HLA alleles for six common HLA gene types (HLA-A, -B, -C, -DQA, DQB, and -DRB) and 14 additional HLA genes (HLA-DOA, -DOB, -DMA, -DMB, -DPA1, -DPB1, -DRQ, -DRB3, -DRB5, -F, -G, -H, -J, and -L). A total of 92,297 structural variants consisting of large deletions, duplications, and inversions, as well as estimated total telomeric content for each participant, were also identified from the WGS data. Figure 8 and Table 5 summarize the genomic data available for the PEGS cohort.

**Fig. 7:**
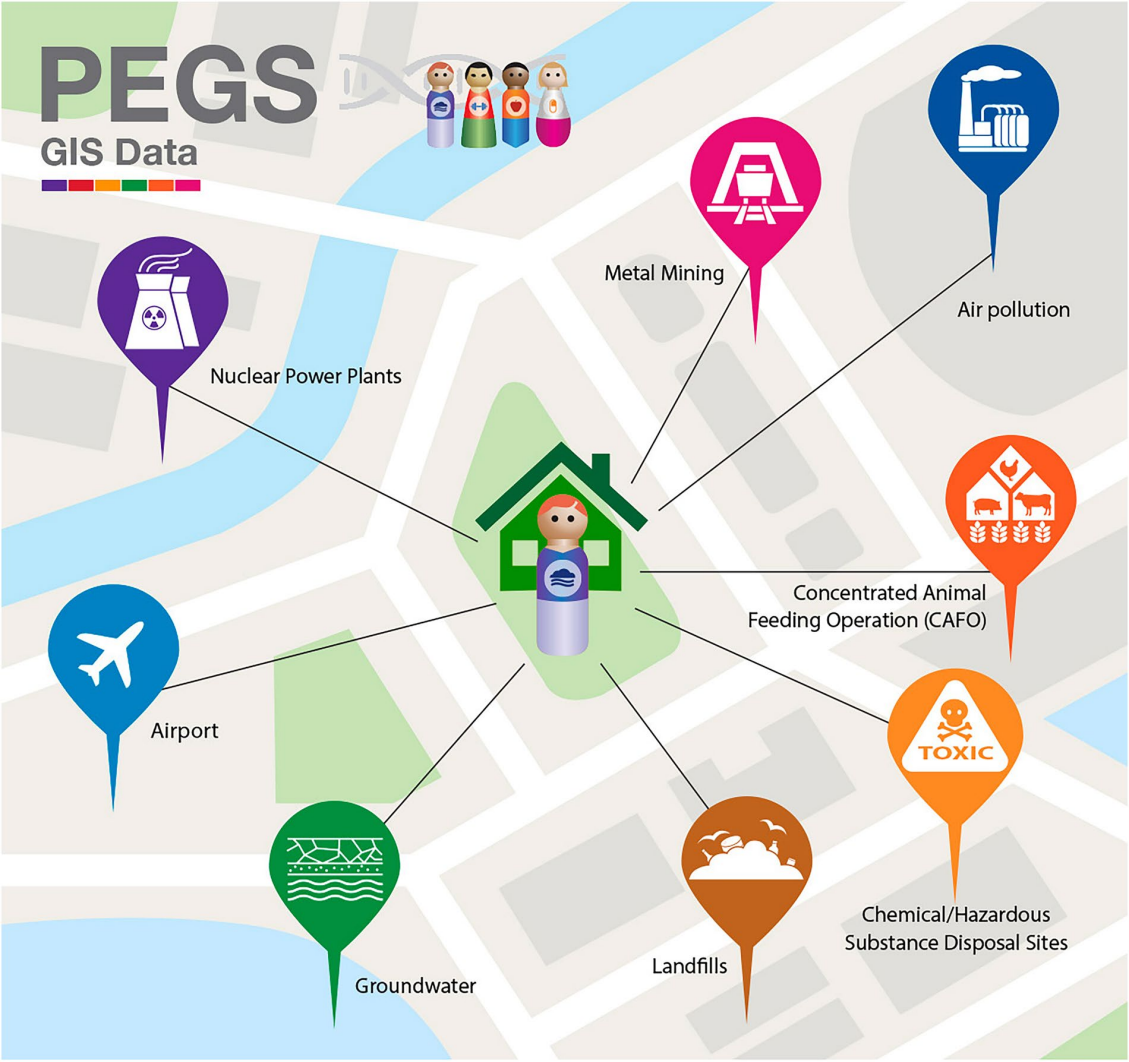
PEGS GIS data. Overview of multi-pollutant point sources used to estimate exposure indicators for PEGS participants using their geocoded addresses. Data reported from PEGS Data Freeze 3.1 created on 6/27/2023.

**Fig. 8:**
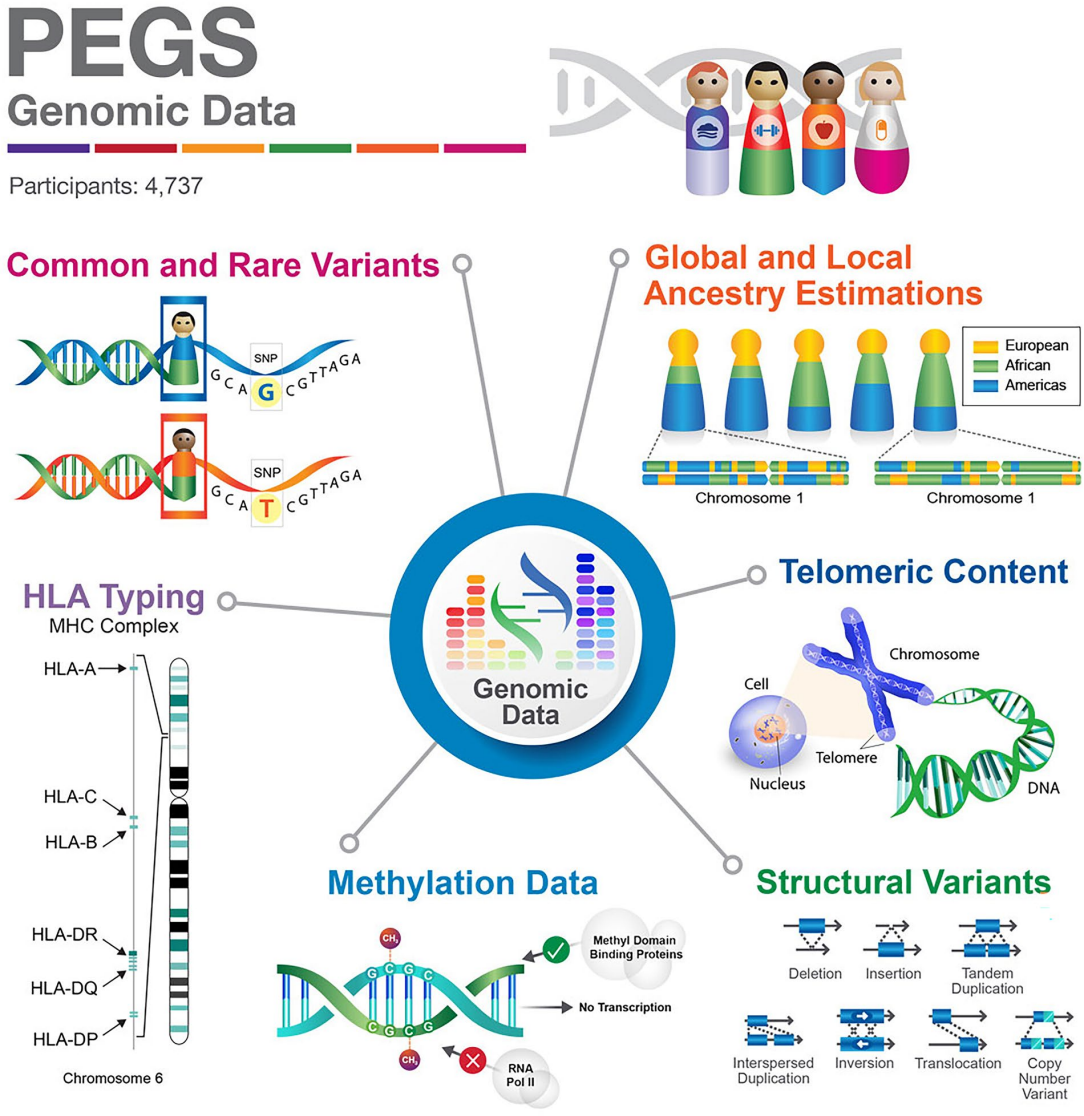
Summary of the genomic data available in the PEGS cohort. Whole-genome sequencing data were used to obtain single-nucleotide variants consisting of common and rare variants, structural variant calls, human leukocyte antigen (HLA) genotypes for 20 HLA genes, aggregate telomeric content estimation, inferred local ancestry per chromosome, and global estimates of percent ancestry. Genome-wide methylation profiling data were obtained using the Infinium MethylationEPIC v1.0 BeadChip Kit. Data reported from PEGS Data Freeze 3.1 created on 6/27/2023.

The Broad Institute sequenced all 4,737 samples on the NovaSeq6000 platform with a target genome-wide read depth of 30× (24 samples per flow cell). The Broad Institute used their gatk4-genome-processing-pipeline version 1.0.0 (github.com/gatk-workflows/gatk4-genome-processing-pipeline) to process reads^7^. Using bwa mem 0.7.15-r1140^8^, the reads were aligned to the hg38 reference genome. Using Picard Tool MarkDuplicates 2.20.4, duplicate fragments were flagged. Finally, using GATK 4.0.10.1 ApplyBQSR, base quality score recalibration was performed.

Variant calling was performed per sample using GATK 3.5.0 HaplotypeCaller with output in g.vcf format, and joint genotyping was performed using GATK 4.1.4.0 GenotypeGvcfs. Subsequently, GATK 4.1.1.0 VariantFiltration was used to apply filtering flags, and GATK 4.1.1.0 ApplyVQSR was utilized for variant quality score recalibration. A “PASS” filter label was assigned to variants with an excess heterozygosity phred score of <  = 54.69 and a VQSLOD score exceeding the threshold of retaining 99.0% of true positive indels and 99.7% of true positive SNPs.

### Quality control analysis for whole-genome sequencing data

Samtools flagstats^9^ was used to determine the total read count and proportion of duplicate fragments. samtools idxstats was used to determine the percentages of reads mapping to the mitochondrial genome and chrY, as well as the ratio of reads mapped to chrX per bp, relative to the genome-wide rate. FastQC 0.11.9 was used to determine the aggregate %GC, separately for mate 1 and mate 2 reads. From the FastQC output, the maximum deviation of mean per-cycle sequence content from genomic background rates of 20% for G/C and 30% for A/T was also determined, excluding the first nine cycles due to the anticipation of large deviations. Per-sample percentages of reads with mapping quality below 5 and 10 were directly derived from the MAPQ field of sample CRAM files. Counts of SNPs and indel variants were derived from preliminary unfiltered GATK joint genotypes called in batches of samples sequenced and processed in tandem. From these preliminary calls, the mean and standard deviation of variant genotype qualities, as well as major allele frequencies, were determined. The major allele frequencies were calculated separately for homozygous and heterozygous genotypes.

Estimates of genomic proportions derived from Asian, Amerindian, European, and African ancestors were determined using ancestry informative markers described by Kosoy *et al*.^10^ and the genotypes of individuals with known ancestry reported in Table S6 of that publication. Genotypes called at these loci for each sample were extracted and independently merged with individuals of known ancestry using PLINK 1.90b4.3^11^ and then analyzed using fastStructure version 1.0^12^ with four specified ancestral populations (-K 4). fastStructure produces genome proportion estimates derived from unlabeled ancestral populations inferred from the given data. Continental associations for each estimated population were determined by calculating the mean proportions separately for individuals of known ancestry and pairing the proportions with a value of 0.9 or higher. In rare cases for which fastStructure’s non-deterministic algorithm did not separate individuals with known ancestry such that each known population could be assigned as described to a single inferred population, the analysis was repeated.

Some individual samples had higher than expected GC content and fractions of reads mapped to chrY. In addition, a small proportion of the genome exceeded the target of 30× coverage. However, these features did not appear to be associated with an increase in novel variant calls or a reduction in genotype quality estimates and hence did not require the removal of any samples. Estimated ancestral genome proportions were manually compared with self-reported race and ethnicity, and ratios of chrX to genomic coverage and fractions of reads mapped to chrY were examined in the context of self-reported sex. These observations indicated no systemic issues of sample swapping or mislabeling.

To ensure that only high-confidence samples are utilized in downstream analyses, we performed further quality control to filter participants as follows. Three samples displayed unusually high read-count variability at loci with heterozygous genotype calls as well as unusually low counts of high-quality variants, indicating that the sequenced sample may have been derived from a mixture of DNA of two individuals. Additionally, visualization of participant read coverage in sex chromosomes revealed four participants with potential aneuploidies. These seven participants were excluded from the WGS dataset. Thus, a sex assignment of “female” was interpreted as “XX” and “male” as “XY”. To mitigate the confounding effects of unknown familial relationships within the cohort, relatedness was assessed using KING 2.2.5, and an additional 120 participants were excluded to ensure the dataset contained no second-degree or closer relatives. This analysis also revealed three pairs of duplicate biological samples for which true participant identities were unclear. The three participants initially retained during relatedness filtering were thus excluded to arrive at the final count of 4,607 participants with WGS data after quality control and filtering.

### Local ancestry estimation

After haplotype phasing using SHAPEIT 4.2.1^13^, RFMix version 2^14^ was used to estimate local ancestry information from WGS data for 29,062,806 harmonized variants in the 4,607 PEGS participants remaining after quality control. Variants genotyped in both the reference panel and PEGS were considered. The reference population for haplotype estimation and local ancestry inference was 2,504 unrelated individuals from the 1000 Genomes Project, Phase 3, with sequencing to a targeted depth of 30× ^15^. Based on 1000 Genomes, each genomic position was classified by five continental super-populations: AFR (African), AMR (Admixed American), EAS (East Asian), EUR (European), and SAS (South Asian)^15^. Each genomic position had two assignments, one for each strand. Genome-wide ancestral similarity proportions were calculated for each participant based on the 29,062,806 assignments.

### Polygenic scores

Polygenic scores (PGS) for PEGS participants with WGS data were computed using data and metadata from the Polygenic Score Catalog^16,17^. To maximize the variants available for PGS calculation and ensure homogeneity across participants, we performed genome-wide imputation via the TOPMed imputation server^18^ (TIS) using Minimac4 and the TOPMed r2 reference panel. After quality control, 4,600 participants with 2,553,563 variants had eligible data for imputation after removing indels, variants with a call rate < 90%, and variants excluded based on the McCarthy Group tools using the TOPMed Freeze 5 reference panel and pruning to LD r^2^ < 0.8 (due to TIS upload size restrictions). Imputed variants with imputation quality Rsq > 0.3 were retained for analysis. PGS and their metadata were downloaded from the Polygenic Score Catalog. Data were harmonized using measures of variant and allele harmonization, including assignment of rsID (where possible) from the PGS Catalog, to allow the creation of files for builds 37 and 38 (via liftover). The PGS Catalog model submission requires the following variant-related fields: chromosome, position, effect allele, and weight, and the non-effect (or “other”) allele is “strongly recommended”. Absence of the “other” allele field can lead to ambiguity with respect to strand and/or multiallelic variants and thus the PGS Catalog data harmonization process attempts to augment this information when missing. To avoid the issue of inconsistency in encoding the number of risk variants on the X chromosome for males vs. females, we restricted to autosomes for PGS calculations.

PGS were computed for PEGS participants from each PGS model using the PLINK 2.0 –score function^19^. Prior to computing scores, we removed palindromic (i.e., A/T or C/G) SNPs and harmonized the PEGS genetic data to match the PGS model effect allele. For each PGS model, we retained the total number of variants in the model, the number excluded, and the number used to create the PGS. Of the 3,490 scores in the PGS catalog (as of May 2023), we excluded 460 scores because less than 70% of the genetic variants were available in the imputed genotype data for PEGS participants. Thus, we were able to compute 3,030 PGS for 4,600 participants. A detailed description of PGS computation is provided in Schaid *et al*.^20^.

### DNA methylation profiling

Genome-wide methylation profiling was collected using the Infinium MethylationEPIC v1.0 BeadChip Kit for 4,724 PEGS participants with available WGS data. The EPIC chip targets 866,297 CpG sites in the most biologically significant regions of the human methylome. After quality control for samples and CpG sites, DNA methylation profile data were available for 826,286 CpG sites for 4,260 participants.

Genomic DNA was extracted from aliquots of whole blood using an automated system (Autopure LS, Gentra Systems) in the NIEHS Molecular Genetics Core Facility or using DNAQuik at BioServe Biotechnologies Ltd. (Beltsville, MD). One microgram of DNA from each participant was bisulfite-converted in 96-well plates using the EZ DNA Methylation Kit (Zymo Research, Orange County, CA), and methylation analysis was conducted at the NCI Center for Cancer Research genomics core center. Samples were tested for completion of bisulfite conversion, and converted DNA was analyzed on Illumina Human MethylationEPIC arrays following the manufacturer’s protocol. The arrays were analyzed with high-throughput robotics to minimize batch effects.

R software package ENmix was employed to perform data quality control and preprocessing, including ENmix background correction, RELIC dye bias correction, separate quantile normalization on methylated and unmethylated intensity values for type I and type II probes, and regression on correlated probes (RCP) for probe type bias adjustment. A total of 480 samples were excluded because of a low-quality CpG value > 5% or bisulfite intensity value < 4350. Additionally, 40,011 CpG sites with >5% low-quality data were excluded. Low-quality data were defined as detection *P* value > 10^−6^ or number of beads < 3.

### Geographic information systems data

In 2020, geocoding was performed by assigning geographic coordinates to participant addresses, enabling proximity and grid-based analysis using distance to contaminant sources and area-level air pollutant concentrations as surrogates for exposure. For each participant, mapping was completed for five participant-provided addresses: at initial enrollment, at completion of the Health and Exposure Survey, at completion of the External Exposome Survey, the longest-lived childhood address, and the longest-lived adulthood address from the External Exposome Survey. The proximity of participants’ geocoded addresses to a variety of multi-pollutant point sources was calculated using publicly available data from federal and state regulatory agencies and then used to estimate exposure indicators. Figure 7 is an overview of the point sources used to estimate exposure indicators based on the geocoded addresses.

Geocoding was performed for five participant-provided addresses: (1) address at initial enrollment; (2) address at completion of the Health and Exposure Survey; (3) address at completion of the External Exposome Survey; (4) the longest-lived childhood address; and (5) the longest-lived adulthood address. CDC/ATSDR Social Vulnerability Index (SVI) (https://www.atsdr.cdc.gov/placeandhealth/svi/index.html) and Environmental Justice Index (EJI) (https://www.atsdr.cdc.gov/placeandhealth/eji/index.html) values, which summarize multiple social determinants of health (SDOH) and measures of the impacts of environmental injustice on health, were linked at the census-tract level to each geocoded address using the SVI/EJI release year corresponding to the closest available time point. For historical addresses, contemporary SVI/EJI values were used due to limited historical index availability, which may introduce temporal misclassification.

Participants’ ambient environmental exposures were estimated by linking publicly available environmental quality data to their geocoded residential addresses. Geocoding was performed using a three-stage process involving 1) U.S. Census Bureau Geocoding Services, 2) the Texas A&M Geocoding Service, and 3) manual review using Quantum GIS referencing public U.S. Census Bureau TIGER/Line street and Landsat orthoimage data. After cleaning and standardizing addresses (e.g., resolving spelling issues and parsing address components), the U.S. Census Bureau Geocoding service performed a first geocoding pass. Failed geocodes were then submitted to the Texas A&M Geocoding Service, which uses a sophisticated fuzzy-matching algorithm to match addresses typed with errors. The remaining unmatched addresses were geocoded using the manual review process, and a random sample of both hand-geocoded and algorithmically geocoded records was re-coded by a second human geocoder for quality assurance. Table 5 summarizes the geospatial data available for the PEGS cohort, and Table 6 outlines the available geocoding and GIS data linkages.

**Table 6:** Summary of PEGS geospatial data.

Source	Description	Examples
Geocodes (GIS)	Geocoded data from multiple participant-provided addresses at time of initial enrollment, completion of the Health and Exposure Survey, completion of the External Exposome Survey, the longest-lived childhood address, and the longest-lived adulthood address from the External Exposome Survey	Geographic coordinates (latitude and longitude) from multiple participant-provided addresses
Hazards	Exposure estimates computed from Department of Transportation (DOT) data	Information from train tracks, rail depots, and roadways such as total major roadway length and distance to the nearest rail depot
Hazards	Exposure estimates computed from Federal Aviation Administration (FAA) data	Information from aircraft departure and arrival sites (e.g., distance to the nearest airport)
Hazards	Exposure estimates computed from Federal Communications Commission (FCC) data	Information from cellular network towers (e.g., nearest cell tower)
Hazards	Exposure estimates computed from North Carolina Department of Environmental Quality (NCDEQ)	Distance to multi-pollutant point sources such as swine caged feeding operations (CAFOs), hazardous waste sites, hazardous spill sites, EPA superfund sites, and wastewater treatment plant release sites
Hazards	Exposure estimates computed from Nuclear Regulatory Commission (NRC) data	Distance to a nuclear power station
Hazards	Exposure estimates computed from Atmospheric Composition and Analysis Group (ACAG) data	Particulate matter concentrations such as PM2.5 total, PM2.5 sulfate, PM2.5 black carbon, and other
Hazards	Exposure estimates computed from Center for Air, Climate, and Energy Solutions (CACES) data	Concentrations for multiple pollutants such as carbon monoxide, nitrogen dioxide, and ozone concentration
Hazards	Exposure estimates computed from Toxics Release Inventory (TRI) data	Emissions for chemicals of interest such as benzene, ethylbenzene, xylene, and toluene
MERRA-2 data (Earthdata)	Geospatial data linkages from the Modern Era Retrospective Analysis for Research and Applications (MERRA-2) project to assimilate a range of satellite-based environmental observations into a consistent estimate of climate and environmental metrics	Particulate, gas, meteorological, and health-relevant exposure indicators such as dust sedimentation, organic carbon emission bin, SO_2_ biomass burning emissions, and sea-level pressure
Social Vulnerability Index (SVI)	Geospatial data linkages for CDC/ATSDR SVI designed to consistently quantify multiple social determinants of health across the United States over time	Consists of summaries of social determinants of health at the census-tract level including an overall index, four component indexes (socioeconomic status, household characteristics, racial and ethnic minority status, and housing type/transportation), and source variables used to compute each index component (e.g., poverty, education, overcrowding, access to a vehicle)
Environmental Justice Index (EJI)	Geospatial data linkages for CDC/ATSDR EJI containing summaries and ranks of the cumulative impacts of environmental injustice on health at the census-tract level	Ranks for each census tract on 36 environmental, social, and health factors grouped into 10 domains and three overarching modules – environmental burden, social vulnerability, and health vulnerability

Overview of the geocoding and GIS data linkages available for the PEGS cohort. Data reported from PEGS Data Freeze 3.1 created on 6/27/2023.

GIS data linkages were performed using these geocodes and R version 4.0 and Geometry Engine Open Source (GEOS) bindings provided by the sp and sf R packages. For proximity measures (e.g., distance to the nearest hazard), we calculated great-circle distances in meters using the WGS84 spheroid. For point-in-polygon linkages (e.g., linking geocodes to measures computed for census tracts), participants were assigned the value of the containing polygons (e.g., census tract or grid cell) using GEOS spatial indexing in the WGS84 coordinate system. Temporal alignments were chosen based on the availability and richness of source data to support expected study designs. For example, land-use models with many available time points (e.g., Center for Air, Climate, and Energy Solutions (CACES) data) were simplified by averaging the containing grid cells over a long period. For area-based measures (e.g., the SVI) for which few discrete time referents are available, all available time points were linked to each geocoded address.

### De-identification of data

De-identification procedures followed the NIST SP 800-122 Guide to Protecting the Confidentiality of Personally Identifiable Information (PII). All direct identifiers were removed prior to data sharing. Dates were generalized to year-only format, and residential address data were converted to geocoded coordinates for internal linkage and then removed from shared datasets. Geographic information provided to approved users is limited to derived exposure metrics and census-tract–level variables. Shared datasets were reviewed to ensure compliance with HIPAA Safe Harbor and Expert Determination standards to minimize re-identification risk.

## Data Record

PEGS comprises a compatible, multi-dimensional collection of datasets in consistent and programmatically extractable formats, as shown in Fig. 2. Survey-based data on exposures and medication, geospatial data, and genomic data are available for PEGS participants. Table 5 outlines the data components of PEGS, including sample sizes for PEGS Data Freeze 3.1. Thorough quality control was conducted to ensure consistent formatting, nomenclature, and data encoding. Metadata were added to each PEGS data component to specify the data format, data encoding, special codes, descriptions, and structure of survey questions to enable the identification of nested questions. There is extensive documentation for each PEGS data component describing the methods used to create the data and details of the variables. PEGS data are available for analysis in R (.RData), SAS (.sas7bdat), and text (.csv) file formats. The quality control performed for the data and its availability in multiple formats facilitates consistent, reproducible, and comparable analyses and supports research collaborations. As of June 27, 2023, PEGS Data Freeze 3.1 is the most recent version released. PEGS data are updated quarterly with data from additional participants, updated data from existing participants, data on new variables, and additional data components.

## Technical Validation

A growing number of projects have used PEGS data. Data have been used to build predictive models for type 2 diabetes^21^, discover gene-environment interactions with immune-mediated disorders^22^, examine exposome-wide associations for cardiovascular diseases^23^, conduct association mapping of the HLA region in asthma^24^, and examine air pollution mixtures and autoimmune skin disease risk^25^. Additionally, several studies have called back participants to collect data to evaluate specific hypotheses. From its inception, a goal of PEGS has been to assist scientists and other investigators by identifying cases and controls for call-back and additional interviews or in-person studies. This has enabled studies investigating the effects of aging and ultraviolet light on somatic mutations that facilitate genome instability and carcinogenesis^26^. Further, these data have been combined with cell-based and mouse studies to uncover the role of a key innate immune receptor in asthma^27^.

## Usage Notes

PEGS data can empower research in a broad range of areas. The data are readily available to confirm or replicate discovery results. Investigators can conduct ExWAS in PEGS data to uncover novel associations between endogenous and exogenous exposures and disease risk, in both single- and multi-exposure analyses. In addition, the data can be used to develop polyexposure risk scores and compare their performance to that of traditional polygenic and clinical risk scores for disease risk prediction^21^. The data can also be used to conduct variance decomposition of disease risk to assess the proportion of disease risk that genetics (G), the environment (E), SDOH (S), and their interactions (GxE, GxS, ExS, GxExS) explain. Additionally, PEGS data can be utilized to explore genetic correlations with environmental exposures, including common variants, rare variants, gene-based associations, and pathway-based analyses, to unravel these complex relationships. Further, incorporating geospatial data enables research on how genomics, the environment, and SDOH contribute to disease risk. Geospatial estimates of exposure, including air quality and proximity to pollutant sources, enable the examination of how weather and climate exposures such as extreme heat are related to disease risk and progression.

Given the diversity and dimensionality of PEGS data, this resource can serve as a testing ground for innovative statistical methods. For example, PEGS data was used in a Sage Bionetworks DREAM Challenge (https://dreamchallenges.org/). DREAM Challenges are collaborative, competitive scientific initiatives that bring together researchers, data scientists, and experts from various fields to address complex biomedical and healthcare problems. Participants are tasked with developing innovative computational models and solutions to solve specific challenges in areas such as disease classification.

NIEHS continues to expand its investment in PEGS through ongoing efforts. Work began in 2019 to link PEGS data with electronic health records (EHRs) of patients of the Duke University Health System and UNC Health at the University of North Carolina Chapel Hill. Linking EHRs for consenting participants will enable the integration of temporal health and disease data and clinical information on multi-dimensional phenotypes, such as International Classification of Diseases (ICD) codes, laboratory data, images, and vital signs. A large call-back study is in progress to collect a range of biospecimens, including blood, urine, stool, saliva, nasal cells, hair, nail clippings, baby teeth, and household dust.

A major goal of PEGS is supporting collaborative research by sharing data with researchers engaged in collaborative projects. Information about submitting proposals for collaborative research is available at: https://www.niehs.nih.gov/research/atniehs/labs/crb/studies/pegs/collaboration/proposal. Several available tools enable users to explore PEGS data and results from analyses conducted in the data. The Informatics for Integrating Biology and the Bedside (i2b2) Query and Analysis Tool facilitates the design and feasibility assessment of potential analyses by allowing researchers to explore aggregated PEGS data^28^. The i2b2 Query and Analysis Tool provides approved users with easy access to explore basic statistics from de-identified and aggregated PEGS data by building customizable queries to display tabular and graphical summaries. The PEGS Explorer web application (https://pegsexplorer.niehs.nih.gov/) was developed to share published results of ExWAS conducted in PEGS data and enable the exploration of rigorously calculated exposure correlations^29,30^. Through globe visualizations, PEGS Explorer users can explore and visualize correlations between exposures associated with common, complex diseases.

Ongoing work in data analysis is focused on building analysis pipelines and workflows to enable efficient, insightful, and collaborative research and ensure consistent, reproducible, and comparable analyses.

## Data Availability

PEGS Data Freeze 3.1 is deposited in a controlled-access data repository hosted by the NIEHS. The repository supports secure, independent download and local analysis of approved data by authorized users. The deposited datasets include de-identified survey data, derived exposure and geospatial metrics, whole-genome sequencing variant files, DNA methylation beta values, polygenic scores, and associated metadata and documentation. Direct identifiers, protected health information, and precise residential address data are not included in shared datasets. Access to PEGS data is available to qualified academic, governmental, and commercial researchers through an application process. Applications are reviewed by the PEGS Executive Leadership Committee using predefined criteria, including scientific validity, consistency with participant consent and NIH IRB approvals, and the applicant’s ability to comply with data security requirements. Approval does not require collaboration with PEGS investigators, and approved users may analyze the data independently. Data access is granted following execution of a Data Use Agreement that specifies permitted uses, data protection requirements, and reporting obligations. Information on the application process and a public copy of the PEGS Data Use Agreement are available at: https://www.niehs.nih.gov/research/atniehs/labs/crb/studies/pegs/collaboration/guidelines.

## Code availability

Standard open-source packages were used to generate the PEGS data as described in the Methods section. The code pipelines for data generation are available at https://github.com/fsakhtari/PEGS_common/blob/master/pegs_common_utils.R.
